# Supplementary material for: Hepcidin in Children and Adults with Acute Leukemia or Undergoing Hematopoietic Cell Transplantation: A Systematic Review
Source: Cancers (Basel). 2022 Oct 8;14(19):4936. doi: 10.3390/cancers14194936 (PMC9561996; doi:10.3390/cancers14194936)
Supplement: Supplementary file 1 [file cancers-14-04936-s001.zip › cancers-1830889-supplementary.pdf]

Supplementary material

Table S1. Preferred Reporting Items for Systematic Reviews and Meta-Analyses (PRISMA) checklist.

Table S2. PubMed search strategy.

Table S3. A complete list of excluded studies along with reasons for exclusion.

Table S4. The Newcastle-Ottawa Scale (NOS) for case-control studies.

Table S5. The Newcastle-Ottawa Scale (NOS) for cohort studies.

Table S6. The Newcastle-Ottawa Scale (NOS) for cross-sectional studies.

Table S1. Preferred Reporting Items for Systematic Reviews and Meta-Analyses (PRISMA) checklist.

Section/topic	#	Checklist item	Reported on page #
**TITLE**			
**Title**	1	Identify the report as a systematic review, meta-analysis, or both.	**1**
**ABSTRACT**			
**Structured summary**	2	Provide a structured summary including, as applicable: background; objectives; data sources; study eligibility criteria, participants, and interventions; study appraisal and synthesis methods; results; limitations; conclusions and implications of key findings; systematic review registration number.	**1**
**INTRODUCTION**			
**Rationale**	3	Describe the rationale for the review in the context of what is already known.	**1**
**Objectives**	4	Provide an explicit statement of questions being addressed with reference to participants, interventions, comparisons, outcomes, and study design (PICOS).	**1**
**METHODS**			
**Protocol and registration**	5	Indicate if a review protocol exists, if and where it can be accessed (e.g., Web address), and, if available, provide registration information including registration number.	**2**
**Eligibility criteria**	6	Specify study characteristics (e.g., PICOS, length of follow-up) and report characteristics (e.g., years considered, language, publication status) used as criteria for eligibility, giving rationale.	**3**
**Information sources**	7	Describe all information sources (e.g., databases with dates of coverage, contact with study authors to identify additional studies) in the search and date last searched.	**2-3**
**Search**	8	Present full electronic search strategy for at least one database, including any limits used, such that it could be repeated.	**Table S2**
**Study selection**	9	State the process for selecting studies (i.e., screening, eligibility, included in systematic review, and, if applicable, included in the meta-analysis).	**3**
**Data collection process**	10	Describe method of data extraction from reports (e.g., piloted forms, independently, in duplicate) and any processes for obtaining and confirming data from investigators.	**3**

**Data items**	11	List and define all variables for which data were sought (e.g., PICOS, funding sources) and any assumptions and simplifications made.	**3**
**Risk of bias in individual studies**	12	Describe methods used for assessing risk of bias of individual studies (including specification of whether this was done at the study or outcome level), and how this information is to be used in any data synthesis.	**4**
**Summary measures**	13	State the principal summary measures (e.g., risk ratio, difference in means).	**N/A**
**Synthesis of results**	14	Describe the methods of handling data and combining results of studies, if done, including measures of consistency (e.g., I^2) for each meta-analysis.	**N/A**
**Risk of bias across studies**	15	Specify any assessment of risk of bias that may affect the cumulative evidence (e.g., publication bias, selective reporting within studies).	**N/A**
**Additional analyses**	16	Describe methods of additional analyses (e.g., sensitivity or subgroup analyses, meta-regression), if done, indicating which were pre-specified.	**N/A**
**RESULTS**			
**Study selection**	17	Give numbers of studies screened, assessed for eligibility, and included in the review, with reasons for exclusions at each stage, ideally with a flow diagram.	**4**
**Study characteristics**	18	For each study, present characteristics for which data were extracted (e.g., study size, PICOS, follow-up period) and provide the citations.	**5**
**Risk of bias within studies**	19	Present data on risk of bias of each study and, if available, any outcome level assessment (see item 12).	**19**
**Results of individual studies**	20	For all outcomes considered (benefits or harms), present, for each study: (a) simple summary data for each intervention group (b) effect estimates and confidence intervals, ideally with a forest plot.	**N/A**
**Synthesis of results**	21	Present results of each meta-analysis done, including confidence intervals and measures of consistency.	**N/A**
**Risk of bias across studies**	22	Present results of any assessment of risk of bias across studies (see Item 15).	**N/A**
**Additional analysis**	23	Give results of additional analyses, if done (e.g., sensitivity or subgroup analyses, meta-regression [see Item 16]).	**N/A**

**DISCUSSION**			
**Summary of evidence**	24	Summarize the main findings including the strength of evidence for each main outcome; consider their relevance to key groups (e.g., healthcare providers, users, and policy makers).	**21**
**Limitations**	25	Discuss limitations at study and outcome level (e.g., risk of bias), and at review-level (e.g., incomplete retrieval of identified research, reporting bias).	**22**
**Conclusions**	26	Provide a general interpretation of the results in the context of other evidence, and implications for future research.	**22-23**
**FUNDING**			
**Funding**	27	Describe sources of funding for the systematic review and other support (e.g., supply of data); role of funders for the systematic review.	**23**

Abbreviations: N/A = not applicable.

Table S2. PubMed search strategy.

A PubMed search was conducted on the 31st of March, 2022.

N	Search	Search details	Records
**1**	hepcidin AND leukemia	("hepcidin s"[All Fields] OR "hepcidine"[All Fields] OR "hepcidins"[MeSH Terms] OR "hepcidins"[All Fields] OR "hepcidin"[All Fields]) AND ("leukaemia"[All Fields] OR "leukemia"[MeSH Terms] OR "leukemia"[All Fields] OR "leukaemias"[All Fields] OR "leukemias"[All Fields] OR "leukemia s"[All Fields])	34
**2**	hepcidin AND hematopoietic stem cell transplantation	("hepcidin s"[All Fields] OR "hepcidine"[All Fields] OR "hepcidins"[MeSH Terms] OR "hepcidins"[All Fields] OR "hepcidin"[All Fields]) AND ("haematopoietic stem cell transplantation"[All Fields] OR "hematopoietic stem cell transplantation"[MeSH Terms] OR ("hematopoietic"[All Fields] AND "stem"[All Fields] AND "cell"[All Fields] AND "transplantation"[All Fields]) OR "hematopoietic stem cell transplantation"[All Fields])	28

Table S3. A complete list of excluded studies along with reasons for exclusion.

Reasons	Number of papers	References
**Articles irrelevant to the current study subject**	45	Vadhan-Raj S, Abonour R, Goldman JW, et al. A first-in-human phase 1 study of a hepcidin monoclonal antibody, LY2787106, in cancer-associated anemia. J Hematol Oncol. 2017;10(1):73.<br><br>Hedenus M, Birgegård G, Näsman P, et al. Addition of intravenous iron to epoetin beta increases hemoglobin response and decreases epoetin dose requirement in anemic patients with lymphoproliferative malignancies: a randomized multicenter study. Leukemia. 2007;21(4):627-632.<br><br>Ihlow J, Gross S, Sick A, et al. AML: high serum ferritin at initial diagnosis has a negative impact on long-term survival. Leuk Lymphoma. 2019;60(1):69-77.<br><br>Ashraf S, Hamid A, Qamar S, Pal HS, Qurat-Ul-Ain, Rehman S. Are Peripheral Blood Counts Predictor of Bone Marrow Infiltration and Hypocellularity in Malignant Neoplasms?. J Coll Physicians Surg Pak. 2021;31(1):79-82.<br><br>Olcay L, Hazirolan T, Yildirmak Y, et al. Biochemical, radiologic, ultrastructural, and genetic evaluation of iron overload in acute leukemia and iron-chelation therapy. J Pediatr Hematol Oncol. 2014;36(4):281-292.<br><br>Haschka D, Petzer V, Kocher F, et al. Classical and intermediate monocytes scavenge non-transferrin-bound iron and damaged erythrocytes. JCI Insight. 2019;4(8):e98867.<br><br>Israel A, Bornstein G, Gilad L, et al. Clinical and prognostic significance of elevated ferritin levels in hospitalised adults. Postgrad

Med J. 2021;postgradmedj-2021-139832.

Tachibana T, Tanaka M, Numata A, et al. Clinical significance of pre- and 1-year post-transplant serum ferritin among adult transplant recipients. Leuk Lymphoma. 2014;55(6):1350-1356.

Sato T, Ichinohe T, Kanda J, et al. Clinical significance of subcategory and severity of chronic graft-versus-host disease evaluated by National Institutes of Health consensus criteria. Int J Hematol. 2011;93(4):532-541.

Beguin Y, Maertens J, De Prijck B, et al. Darbepoetin-alfa and intravenous iron administration after autologous hematopoietic stem cell transplantation: a prospective multicenter randomized trial. Am J Hematol. 2013;88(12):990-996.

Visani G, Guiducci B, Giardini C, Loscocco F, Ricciardi T, Isidori A. Deferasirox improves hematopoiesis after allogeneic hematopoietic SCT. Bone Marrow Transplant. 2014;49(4):585-587.

Li B, Espósito BP, Wang S, et al. Desferrioxamine-caffeine shows improved efficacy in chelating iron and depleting cancer stem cells. J Trace Elem Med Biol. 2019;52:232-238.

Kim SE, Kim UJ, Jang MO, et al. Diagnostic use of serum ferritin levels to differentiate infectious and noninfectious diseases in patients with fever of unknown origin. Dis Markers. 2013;34(3):211-218.

Jullien M, Orvain C, Berceanu A, et al. Early Post-Transplantation Serum Ferritin Level Predicts Survival in Recipients of Haploidentical Stem Cell Transplantation Using Post-Transplantation Cyclophosphamide as Graft-versus-Host Disease Prophylaxis. Transplant Cell Ther. 2021;27(10):861.e1-861.e7.

Luo T, Gao J, Lin N, Wang J. Effects of Two Kinds of Iron

Nanoparticles as Reactive Oxygen Species Inducer and Scavenger on the Transcriptomic Profiles of Two Human Leukemia Cells with Different Stemness. Nanomaterials (Basel). 2020;10(10):1951.

Nakamaki T, Kawabata H, Saito B, et al. Elevated levels of transferrin receptor 2 mRNA, not transferrin receptor 1 mRNA, are associated with increased survival in acute myeloid leukaemia. Br J Haematol. 2004;125(1):42-49.

Petzer V, Wermke M, Tymoszuk P, et al. Enhanced labile plasma iron in hematopoietic stem cell transplanted patients promotes *Aspergillus* outgrowth. Blood Adv. 2019;3(11):1695-1700.

Trujillo-Alonso V, Pratt EC, Zong H, et al. FDA-approved ferumoxytol displays anti-leukaemia efficacy against cells with low ferroportin levels. Nat Nanotechnol. 2019;14(6):616-622.

Dorak MT, Mackay RK, Relton CL, Worwood M, Parker L, Hall AG. Hereditary hemochromatosis gene (HFE) variants are associated with birth weight and childhood leukemia risk. Pediatr Blood Cancer. 2009;53(7):1242-1248.

El-Rashedi FH, El-Hawy MA, El-Hefnawy SM, Mohammed MM. HFE gene mutation and iron overload in Egyptian pediatric acute lymphoblastic leukemia survivors: a single-center study. Hematology. 2017;22(7):398-404.

Kurokawa M, Nishiyama K, Koga Y, et al. Hyperferritinemia and acute kidney injury in pediatric patients receiving allogeneic hematopoietic cell transplantation. Pediatr Nephrol. 2020;35(10):1977-1984.

Li S, Bian H, Cao Y, et al. Identification of novel lncRNAs involved in the pathogenesis of childhood acute lymphoblastic leukemia. Oncol

Lett. 2019;17(2):2081-2090.

Zhao Y, Gao F, Shi J, et al. Incidence, Risk Factors, and Outcomes of Primary Poor Graft Function after Allogeneic Hematopoietic Stem Cell Transplantation. Biol Blood Marrow Transplant. 2019;25(9):1898-1907.

Panch SR, Yau YY, West K, Diggs K, Sweigart T, Leitman SF. Initial serum ferritin predicts number of therapeutic phlebotomies to iron depletion in secondary iron overload. Transfusion. 2015;55(3):611-622.

Steeghs EMP, Jerchel IS, de Goffau-Nobel W, et al. JAK2 aberrations in childhood B-cell precursor acute lymphoblastic leukemia. Oncotarget. 2017;8(52):89923-89938.

Gasparetto M, Pei S, Minhajuddin M, Stevens B, Smith CA, Seligman P. Low ferroportin expression in AML is correlated with good risk cytogenetics, improved outcomes and increased sensitivity to chemotherapy. Leuk Res. 2019;80:1-10.

Kaneko Y, Miyajima H, Piperno A, et al. Measurement of serum hepcidin-25 levels as a potential test for diagnosing hemochromatosis and related disorders. J Gastroenterol. 2010;45(11):1163-1171.

Karalexi MA, Pourtsidis A, Panagopoulou P, et al. Overall and event-free survival of childhood lymphoma in Greece: analysis of harmonized clinical data over a 24-year active registration period. Leuk Lymphoma. 2021;62(9):2107-2119.

Brenner AK, Tvedt TH, Nepstad I, et al. Patients with acute myeloid leukemia can be subclassified based on the constitutive cytokine release of the leukemic cells; the possible clinical relevance and the importance of cellular iron metabolism. Expert Opin Ther Targets.

2017;21(4):357-369.

Kansagra KA, Parmar D, Jani RH, et al. Phase I Clinical Study of ZYAN1, A Novel Prolyl-Hydroxylase (PHD) Inhibitor to Evaluate the Safety, Tolerability, and Pharmacokinetics Following Oral Administration in Healthy Volunteers. Clin Pharmacokinet. 2018;57(1):87-102.

Nourkami-Tutdibi N, Graf N, Beier R, Zemlin M, Tutdibi E. Plasma levels of osteopontin from birth to adulthood. Pediatr Blood Cancer. 2020;67(7):e28272.

Hegab HM, El-Ghammaz AMS, El-Razzaz MK, Helal RAA. Prognostic Impact of Serum Growth Differentiation Factor 15 Level in Acute Myeloid Leukemia Patients. Indian J Hematol Blood Transfus. 2021;37(1):37-44.

Rapido F, Brittenham GM, Bandyopadhyay S, et al. Prolonged red cell storage before transfusion increases extravascular hemolysis. J Clin Invest. 2017;127(1):375-382.

Wang R, Huang A, Chen Q, et al. Pulmonary Infection Within 100 Days After Transplantation Impaired Platelet Recovery in Patients with Hematologic Malignancies: A Propensity-Score-Matched Analysis. Ann Transplant. 2019;24:541-552.

Teixeira C, Barbot J, Freitas MI. Reference values for reticulocyte parameters and hypochromic RBC in healthy children. Int J Lab Hematol. 2015;37(5):626-630.

Wardhani SO, Oehadian A. Reticulocyte hemoglobin equivalent (Ret-he) as measurement of bone marrow iron storage. UHOD - Uluslararasi Hematoloji-Onkoloji Dergisi. 2021;31(2):79-84.

Tachibana T, Takasaki H, Tanaka M, et al. Serum ferritin and disease

status at transplantation predict the outcome of allo-SCT in patients with AML or myelodysplastic syndrome. Bone Marrow Transplant. 2011;46(1):150-151.

Papageorgiou SG, Kotsianidis I, Bouchla A, et al. Serum ferritin and ECOG performance status predict the response and improve the prognostic value of IPSS or IPSS-R in patients with high-risk myelodysplastic syndromes and oligoblastic acute myeloid leukemia treated with 5-azacytidine: a retrospective analysis of the Hellenic national registry of myelodysplastic and hypoplastic syndromes. Ther Adv Hematol. 2020;11:2040620720966121.

Kossiva L, Gourgiotis DI, Tsentidis C, et al. Serum hepcidin and ferritin to iron ratio in evaluation of bacterial versus viral infections in children: a single-center study. Pediatr Infect Dis J. 2012;31(8):795-798.

Jaspers A, Baron F, Willems E, et al. Serum hepcidin following autologous hematopoietic cell transplantation: an illustration of the interplay of iron status, erythropoiesis and inflammation. Haematologica. 2014;99(3):e35-e37.

Steensma DP, Sasu BJ, Sloan JA, Tomita DK, Loprinzi CL. Serum hepcidin levels predict response to intravenous iron and darbepoetin in chemotherapy-associated anemia. Blood. 2015;125(23):3669-3671.

Al-Darwish M, Farhan N, Al-Jebreen A, et al. The contribution of multiple packed red blood cell transfusions toward cardiac and liver dysfunction in pediatric patients with acute myeloid leukemia. Leuk Lymphoma. 2016;57(10):2472-2475.

Wang L, Li X, Mu Y, et al. The iron chelator desferrioxamine synergizes with chemotherapy for cancer treatment. J Trace Elem Med Biol. 2019;56:131-138.

		Gutiérrez L, House MJ, Vasavda N, et al. Tissue Iron Distribution Assessed by MRI in Patients with Iron Loading Anemias. PLoS One. 2015;10(9):e0139220.<br>Salamin O, Mignot J, Kuuranne T, Saugy M, Leuenberger N. Transcriptomic biomarkers of altered erythropoiesis to detect autologous blood transfusion. Drug Test Anal. 2018;10(3):604-608.
**Review articles**	42	Lindo L, Wilkinson LH, Hay KA. Befriending the Hostile Tumor Microenvironment in CAR T-Cell Therapy. Front Immunol. 2021;11:618387.<br>Kohgo Y, Ikuta K, Ohtake T, Torimoto Y, Kato J. Body iron metabolism and pathophysiology of iron overload. Int J Hematol. 2008;88(1):7-15.<br>Wood LJ, Nail LM, Gilster A, Winters KA, Elsea CR. Cancer chemotherapy-related symptoms: evidence to suggest a role for proinflammatory cytokines. Oncol Nurs Forum. 2006;33(3):535-542.<br>Raza M, Chakraborty S, Choudhury M, Ghosh PC, Nag A. Cellular iron homeostasis and therapeutic implications of iron chelators in cancer. Curr Pharm Biotechnol. 2014;15(12):1125-1140.<br>Shores DR, Everett AD. Children as Biomarker Orphans: Progress in the Field of Pediatric Biomarkers. J Pediatr. 2018;193:14-20.e31.<br>Shander A, Sazama K. Clinical consequences of iron overload from chronic red blood cell transfusions, its diagnosis, and its management by chelation therapy. Transfusion. 2010;50(5):1144-1155.<br>Juskevicius R, Thompson MA, Shaver A, Head D. Clinical presentation, diagnosis, and classification of acute myeloid leukemia. Hematologic Malignancies. 2021:11-55.

Atilla E, Toprak SK, Demirer T. Current Review of Iron Overload and Related Complications in Hematopoietic Stem Cell Transplantation. Turk J Haematol. 2017;34(1):1-9.

Taher AT, Musallam KM, Hoffbrand AV. Current strategies in the assessment of iron overload. European Journal of Clinical and Medical Oncology. 2011;3(3):30-7.

Vela D, Vela-Gaxha Z. Differential regulation of hepcidin in cancer and non-cancer tissues and its clinical implications. Exp Mol Med. 2018;50(2):e436.

Gamage SMK, Lee KTW, Dissabandara DLO, Lam AK, Gopalan V. Dual role of heme iron in cancer; promotor of carcinogenesis and an inducer of tumour suppression. Exp Mol Pathol. 2021;120:104642.

Zhang J, Liu Y, Li Q, Xu A, Hu Y, Sun C. Ferroptosis in hematological malignancies and its potential network with abnormal tumor metabolism. Biomed Pharmacother. 2022;148:112747.

Grignano E, Birsen R, Chapuis N, Bouscary D. From Iron Chelation to Overload as a Therapeutic Strategy to Induce Ferroptosis in Leukemic Cells. Front Oncol. 2020;10:586530.

Rupolo M, Lleshi A, Cacopardo B, Michieli M, Berretta M. Hematopoietic growth factors support in the elderly cancer patients treated with antiblastic chemotherapy. Anticancer Agents Med Chem. 2013;13(9):1438-1443.

Poli M, Asperti M, Ruzzenenti P, Regoni M, Arosio P. Hepcidin antagonists for potential treatments of disorders with hepcidin excess. Front Pharmacol. 2014;5:86.

Dorak MT, Burnett AK, Worwood M. HFE gene mutations in susceptibility to childhood leukemia: HuGE review. Genet Med.

2005;7(3):159-168.

Wang F, Lv H, Zhao B, et al. Iron and leukemia: new insights for future treatments. J Exp Clin Cancer Res. 2019;38(1):406.

Tanno T, Miller JL. Iron Loading and Overloading due to Ineffective Erythropoiesis. Adv Hematol. 2010;2010:358283.

Kanda J, Kawabata H, Chao NJ. Iron overload and allogeneic hematopoietic stem-cell transplantation. Expert Rev Hematol. 2011;4(1):71-80.

Franke GN, Kubasch AS, Cross M, Vucinic V, Platzbecker U. Iron overload and its impact on outcome of patients with hematological diseases. Mol Aspects Med. 2020;75:100868.

Shander A, Cappellini MD, Goodnough LT. Iron overload and toxicity: the hidden risk of multiple blood transfusions. Vox Sang. 2009;97(3):185-197.

Ozment CP, Turi JL. Iron overload following red blood cell transfusion and its impact on disease severity. Biochim Biophys Acta. 2009;1790(7):694-701.

Deeg HJ, Spaulding E, Shulman HM. Iron overload, hematopoietic cell transplantation, and graft-versus-host disease. Leuk Lymphoma. 2009;50(10):1566-1572.

Fibach E, Rachmilewitz EA. Iron overload in hematological disorders. Presse Med. 2017;46(12 Pt 2):e296-e305.

Majhail NS, Lazarus HM, Burns LJ. Iron overload in hematopoietic cell transplantation. Bone Marrow Transplant. 2008;41(12):997-1003.

Isidori A, Loscocco F, Visani G, et al. Iron Toxicity and Chelation Therapy in Hematopoietic Stem Cell Transplant. Transplant Cell Ther.

2021;27(5):371-379.

Diesch-Furlanetto T, Gabriel M, Zajac-Spychala O, Cattoni A, Hoeben BAW, Balduzzi A. Late Effects After Haematopoietic Stem Cell Transplantation in ALL, Long-Term Follow-Up and Transition: A Step Into Adult Life. Front Pediatr. 2021;9:773895.

Brissot E, Savani BN, Mohty M. Management of high ferritin in long-term survivors after hematopoietic stem cell transplantation. Semin Hematol. 2012;49(1):35-42.

Porter JB, de Witte T, Cappellini MD, Gattermann N. New insights into transfusion-related iron toxicity: Implications for the oncologist. Crit Rev Oncol Hematol. 2016;99:261-271.

Galanello R, Origa R. Once-daily oral deferasirox for the treatment of transfusional iron overload. Expert Rev Clin Pharmacol. 2008;1(2):231-240.

Barton JC. Optimal management strategies for chronic iron overload. Drugs. 2007;67(5):685-700.

Kontoghiorghe CN, Kolnagou A, Kontoghiorghes GJ. Potential clinical applications of chelating drugs in diseases targeting transferrin-bound iron and other metals. Expert Opin Investig Drugs. 2013;22(5):591-618.

Chen S, Wang X, Cui J. Progress research on abnormal iron metabolism of cancer patients and its application. Journal of Jilin University Medicine Edition. 2017;43(1):200-2044.

Glaspy JA. Randomized controlled trials of the erythroid-stimulating agents in cancer patients. Cancer Treat Res. 2011;157:195-215.

Roubinian N, Carson JL. Red Blood Cell Transfusion Strategies in

		Adult and Pediatric Patients with Malignancy. Hematol Oncol Clin North Am. 2016;30(3):529-540.<br>Weber S, Parmon A, Kurrle N, Schnütgen F, Serve H. The Clinical Significance of Iron Overload and Iron Metabolism in Myelodysplastic Syndrome and Acute Myeloid Leukemia. Front Immunol. 2021;11:627662.<br>Sivgin S, Eser B. The management of iron overload in allogeneic hematopoietic stem cell transplant (alloHSCT) recipients: where do we stand?. Ann Hematol. 2013;92(5):577-586.<br>de Witte T. The role of iron in patients after bone marrow transplantation. Blood Rev. 2008;22 Suppl 2:S22-S28.<br>Brissot E, Bernard DG, Loréal O, Brissot P, Troadec MB. Too much iron: A masked foe for leukemias. Blood Rev. 2020;39:100617.<br>Leitch HA, Fibach E, Rachmilewitz E. Toxicity of iron overload and iron overload reduction in the setting of hematopoietic stem cell transplantation for hematologic malignancies. Crit Rev Oncol Hematol. 2017;113:156-170.<br>Shah N, Andrews J, Goodnough LT. Transfusions for anemia in adult and pediatric patients with malignancies. Blood Rev. 2015;29(5):291-299.<br>Tinsley SM, Hoehner-Cooper CM. Transitioning Patients With Iron Overload From Exjade to Jadenu. J Infus Nurs. 2018;41(3):171-175.
**Articles without sufficient data on hepcidin levels**	15	Göker B, Akbiyik M, Gönül O, Güleç Ç, Anak S. Determination of relationship between Infection and Serum Levels of Prohepcidin in Pediatric Patients before and after Bone Marrow Transplantation. Journal of Pediatric Biochemistry. 2015;5(3):94-97.

Wennerås C, Hagberg L, Andersson R, et al. Distinct inflammatory mediator patterns characterize infectious and sterile systemic inflammation in febrile neutropenic hematology patients. PLoS One. 2014;9(3):e92319.

Armand P, Sainvil MM, Kim HT, et al. Does iron overload really matter in stem cell transplantation?. Am J Hematol. 2012;87(6):569-572.

Çelik H, Lindblad KE, Popescu B, et al. Highly multiplexed proteomic assessment of human bone marrow in acute myeloid leukemia. Blood Adv. 2020;4(2):367-379.

Akı SZ, Paşaoğlu H, Yeğin ZA, et al. Impact of prohepcidin levels and iron parameters on early post-transplantation toxicities. Hematology. 2011;16(5):284-290.

Pullarkat V. Iron overload in patients undergoing hematopoietic stem cell transplantation. Adv Hematol. 2010;2010:345756.

Wurschi GW, Mentzel HJ, Herrmann KH, et al. MRI as an alternative to serum ferritin for diagnosis of iron overload in children in the context of immune response after stem cell transplantation. Pediatr Transplant. 2019;23(8):e13583.

Maximova N, Gregori M, Boz G, et al. MRI-based evaluation of multiorgan iron overload is a predictor of adverse outcomes in pediatric patients undergoing allogeneic hematopoietic stem cell transplantation. Oncotarget. 2017;8(45):79650-79661.

Nogai A, Shi Y, Pérez-Hernandez D, et al. Organ siderosis and hemophagocytosis during acute graft-versus-host disease. Haematologica. 2016;101(8):e344-e346.

Tachibana T, Tanaka M, Takasaki H, et al. Pre-SCT serum ferritin is a

		prognostic factor in adult AML, but not ALL. Bone Marrow Transplant. 2011;46(9):1268-1269.<br><br>Sivgin S, Baldane S, Kaynar L, et al. Pretransplant iron overload may be associated with increased risk of invasive fungal pneumonia (IFP) in patients that underwent allogeneic hematopoietic stem cell transplantation (alloHSCT). Transfus Apher Sci. 2013;48(1):103-108.<br><br>Sirvent A, Auquier P, Oudin C, et al. Prevalence and risk factors of iron overload after hematopoietic stem cell transplantation for childhood acute leukemia: a LEA study. Bone Marrow Transplant. 2017;52(1):80-87.<br><br>Amid A, Barrowman N, Vijenthira A, Lesser P, Mandel K, Ramphal R. Risk factors for hyperferritinemia secondary to red blood cell transfusions in pediatric cancer patients. Pediatr Blood Cancer. 2013;60(10):1671-1675.<br><br>Yalaki AI, Kupeli BY, Kupeli S, Bayram I, Dilek O, Yilmaz C. Transfusion associated iron overload in childhood cancer survivors. Cukurova Medical Journal. 2018;43(4):810-815.<br><br>De Bock M, Beguin Y, Leprince P, et al. Comprehensive plasma profiling for the characterization of graft-versus-host disease biomarkers. Talanta. 2014;125:265-275.
**Nonhuman studies**	9	Li X, Xu F, Karoopongse E, et al. Allogeneic transplantation, Fas signaling, and dysregulation of hepcidin. Biol Blood Marrow Transplant. 2013;19(8):1210-1219.<br><br>Krause DS, Fulzele K, Catic A, et al. Differential regulation of myeloid leukemias by the bone marrow microenvironment. Nat Med. 2013;19(11):1513-1517.<br><br>Karoopongse E, Marcondes AM, Yeung C, et al. Disruption of Iron

		Regulation after Radiation and Donor Cell Infusion. Biol Blood Marrow Transplant. 2016;22(7):1173-1181.<br><br>Jazi MS, Mohammadi S, Yazdani Y, Sedighi S, Memarian A, Aghaei M. Effects of valproic acid and pioglitazone on cell cycle progression and proliferation of T-cell acute lymphoblastic leukemia Jurkat cells. Iran J Basic Med Sci. 2016;19(7):779-786.<br><br>Argenziano M, Tortora C, Paola AD, et al. Eltrombopag and its iron chelating properties in pediatric acute myeloid leukemia. Oncotarget. 2021;12(14):1377-1387.<br><br>Stefanova D, Raychev A, Arezes J, et al. Endogenous hepcidin and its agonist mediate resistance to selected infections by clearing non-transferrin-bound iron. Blood. 2017;130(3):245-257.<br><br>Song X, Xie Y, Kang R, et al. FANCD2 protects against bone marrow injury from ferroptosis. Biochem Biophys Res Commun. 2016;480(3):443-449.<br><br>Lopes M, Duarte TL, Teles MJ, et al. Loss of erythroblasts in acute myeloid leukemia causes iron redistribution with clinical implications. Blood Adv. 2021;5(16):3102-3112.<br><br>La Carpia F, Wojczyk BS, Annavajhala MK, et al. Transfusional iron overload and intravenous iron infusions modify the mouse gut microbiota similarly to dietary iron. NPJ Biofilms Microbiomes. 2019;5(1):26.
**Case reports**	1	Balagtas JM, Dahl GV. Therapeutic complications in a patient with high-risk acute lymphoblastic leukemia and undiagnosed hereditary hemochromatosis. Pediatr Blood Cancer. 2012;58(1):101-103.
**Non-English articles**	7	Wollmer E, Neubauer A. Complications after allogeneic bone marrow

		and stem cell transplantation. Internist. 2014;55(5):547-561.<br><br>Chen SA, Zhang WB. Expression Changes of Serum Transferrin Receptor and Its Mechanism in Children with Acute Leukemia. Zhongguo Shi Yan Xue Ye Xue Za Zhi. 2018;26(6):1637-1643.<br><br>Pan LL, Gao J, Chen TT, et al. Expression of transferrin receptor 2 in mononuclear cells from children with acute leukemia. Sichuan Da Xue Xue Bao Yi Xue Ban. 2009;40(1):20-76.<br><br>Makeshova AB, Levina AA, Mamukova YI, Parovichnikova EN, Savchenko VG. Iron metabolism regulatory mechanisms in initial leukocytosis in patients with acute leukemia. Ter Arkh. 2011;83(10):22-27.<br><br>De Soyano AM, Soyano A. Iron overload in hematologic and non-hematologic diseases. A time bomb. Gaceta Medica de Caracas. 2013;121(3):209-224.<br><br>Maas RP, Voets PJ, de Swart L, Swinkels DW. Non-transferrin-bound iron: a promising biomarker in iron overload disorders. Ned Tijdschr Geneeskd. 2013;157(49):A6258.<br><br>Makeshova AB, Levina AA, Mamukova IuI, Melik-Nubarov NS, Tsibul'skaia MM, Savchenko VG. Value of dynamic study of cytokines in serum and leukocytes in patients with acute leukemia. Klin Lab Diagn. 2009;(12):19-24.
**Comments on articles**	4	Leitch HA. Defining clinically relevant measures of iron overload around haemopoietic stem cell transplantation. Lancet Haematol. 2018;5(5):e184-e185.<br><br>Koreth J, Antin JH. Iron overload in hematologic malignancies and outcome of allogeneic hematopoietic stem cell transplantation.

		Haematologica. 2010;95(3):364-366.<br>Pullarkat V. Iron toxicity in hematopoietic stem cell transplantation: Strike while the iron is labile. Acta Haematol. 2014;131(4):220-221.<br>Kamble R, Mims M. Iron-overload in long-term survivors of hematopoietic transplantation. Bone Marrow Transplant. 2006;37(8):805-806.
**Position article and guidelines**	1	Remacha A, Sanz C, Contreras E, et al. Guidelines on haemovigilance of post-transfusional iron overload. Blood Transfus. 2013;11(1):128-139.
**Conference proceeding**	7	Naoum FA, Esposito BP, Ruiz MA, Cancado R, Barros JC. Assessmet of labile plasma iron and hepcidin in patients who undergo hematopoietic stem cell transplantation. Blood. 2014;124(21):4029.<br>Kanda J, Mizumoto C, Kawabata H, et al. Clinical significance of serum hepcidin-25 levels on early treatment-related complications after hematopoietic stem cell transplantation. Blood. 2008;112(11):1160.<br>Arvedson TC, Li H, Rose MJ, Wang O, Sasu BJ. Evaluation of serum hepcidin concentrations in cancer and leukemia patients. JCO. 2008;26(15_suppl):20581-20581.<br>El-Sayed MH, Matter RM, Abdel-Gawad AN, Abdel-Aziz MM, Esmat GE, Mokhtar GM. Hepcidin and Iron Overload in HCV-infected Children with Acute Leukemia. Hepatology. 2013;58(S1):806A.<br>Zaucha-Prazmo A, Gozdzik J, Drabko K, Wozniak M, Kowalczyk JR. Is hepcidin a better marker of iron overload in children treated with hematopoietic stem cell transplantation? Bone Marrow Transplant. 2014;49(1):S372-S373.<br>Kanda J, Mizumoto C, Kawabata H, Tsuchida H, Tomosugi N,

		Uchiyama T. Serum level of hepcidin and erythropoietic activity after hematopoietic stem cell transplantation. Blood. 2007;110(11):3000.<br><br>Liu H-C, Yeh T-C, Hou J-Y, et al. The Dysregulation of Hepcidin-Ferroportin Axis in Childhood Acute Lymphoblastic Leukemia Survivors after Completion of Chemotherapy. Blood. 2020;136(Supplement 1):1.
**Meta-analysis**	1	Garcia-Casal MN, Pasricha SR, Martinez RX, Lopez-Perez L, Peña-Rosas JP. Serum or plasma ferritin concentration as an index of iron deficiency and overload. Cochrane Database Syst Rev. 2021;5(5):CD011817.
**Clinical trials**	2	Jaekel N, Lieder K, Albrecht S, et al. Efficacy and safety of deferasirox in non-thalassemic patients with elevated ferritin levels after allogeneic hematopoietic stem cell transplantation. Bone Marrow Transplant. 2016;51(1):89-95.<br><br>Pirotte M, Fillet M, Seidel L, Jaspers A, Baron F, Beguin Y. Erythroferrone and hepcidin as mediators between erythropoiesis and iron metabolism during allogeneic hematopoietic stem cell transplant. Am J Hematol. 2021;96(10):1275-1286.

Table S4. The Newcastle-Ottawa Scale (NOS) for case-control studies.

Newcastle-Ottawa Scale (NOS)									
	**Selection**				**Comparability**	**Exposure**			
**First author**	Is the case definition adequate?	Representativeness of the cases	Selection of controls	Definition of controls	Comparability of cases and controls on the basis of the design or analysis	Ascertainment of exposure	Same method of ascertainment for cases and controls	Non-response rate	**Total score**
Chen J [34]	★	★	-	★	-	★	★	N/A	**5**
Yavuz G [37]	★	★	-	★	★	★	★	N/A	**6**
Cheng PP [20]	★	★	-	★	★★	★	★	N/A	**7**
Eisfeld AK [32]	★	★	-	★	★★	★	★	N/A	**7**
Kanda J [22]	★	★	-	★	-	★	★	N/A	**5**
Ragab SM [36]	★	★	-	★	★★	★	★	N/A	**7**
Lecka M [21]	★	★	-	★	★★	★	★	N/A	**7**

Abbreviations: N/A = not applicable.

Table S5. The Newcastle-Ottawa Scale (NOS) for cohort studies.

Newcastle-Ottawa Scale (NOS)									
	Selection				Comparability	Exposure			
**First author**	Representativeness of the exposed cohort	Selection of the non exposed cohort	Ascertainment of exposure	Demonstration that outcome of interest was not present at start of study	Comparability of cohorts on the basis of the design or analysis	Assessment of outcome	Was follow-up long enough for outcomes to occur	Adequacy of follow up of cohorts	**Total score**
Kanda J [31]	★	N/A	★	★	N/A	★	★	★	**6**
Wermke M [38]	★	N/A	★	★	N/A	★	★	★	**6**
Sakamoto S [24]	★	N/A	★	★	N/A	★	★	★	**6**
Naoum FA [35]	★	N/A	★	★	N/A	★	★	★	**6**

Abbreviations: N/A = not applicable.

Table S6. The Newcastle-Ottawa Scale (NOS) for cross-sectional studies.

Newcastle-Ottawa Scale (NOS)								
**First author**	**Selection**				**Comparability**	**Exposure**		**Total score**
	Representativeness of the sample	Sample size	Non-respondents	Ascertainment of the exposure (risk factor)	The subjects in different outcome groups are comparable, based on the study design or analysis<br>Confounding factors are controlled	Assessment of outcome	Statistical test	
Wande N [39]	★	-	N/A	★	★★	★★	-	**6**
Armand P [33]	★	-	N/A	★	★★	★★	-	**6**

Abbreviations: N/A = not applicable.
